# Is it more dangerous to perform inadequate packing?

**DOI:** 10.1186/1749-7922-3-1

**Published:** 2008-01-14

**Authors:** Unal Aydin, Pinar Yazici, Murat Zeytunlu, Ahmet Coker

**Affiliations:** 1 Ege University School of Medicine, Department of General Surgery, Izmir, Turkey

## Abstract

Peri-hepatic packing procedure, which is the basic damage control technique for the treatment of hepatic hemorrhage, is one of the cornerstones of the surgical strategy for abdominal trauma. The purpose of this study was to evaluate the efficacy of the perihepatic packing procedure by comparing the outcomes of appropriately and inappropriately performed interventions. Trauma patients with liver injury were retrospectively evaluated. The patients who had undergone adequate packing were classified as Group A, and the patients who had undergone inappropriate packing, as Group B. Over a five-year period, nineteen patients underwent perihepatic packing. Thirteen of these patients were referred by other hospitals. Of 13 patients, 9 with inappropriate packing procedure due to insertion of intraabdominal drainage catheter (n=4) and underpacking (n=5) were evaluated in Group B, and the others (n=10) with adequate packing were assessed in Group A. Mean 3 units of blood were transfused in Group A and unpacking procedure was performed in the 24^th ^hour. Only 3 (30%) patients required segment resection with homeostasis, and the mortality rate was 20% (2/10 patients). In Group B, 4 patients required repacking in the first 6 hrs. Mean 8 units of blood were transfused until unpacking procedure. The mortality rate was 44% (4/9 patients). The length of intensive care unit stay and requirement of blood transfusion were statistically significantly lower in Group A (p < 0.05). The mortality rate of this group was also lower. However, the difference between the groups for mortality rates was not statistically significant. This study emphasizes that efficacy of the procedure is one of the determinants that affects the results, and inadequate or inappropriate packing may easily result in poor outcome.

## Background

Although small wounds of the liver parenchyma can be managed with electrocautery or simple suturing and hemodynamically stable patients, mostly with low-grade hepatic injuries due to blunt injury, can be managed nonoperatively, the treatment strategy of the patients sustaining major hepatic trauma is still controversial. Particularly deeper lacerations of the hepatic tissue are challenging for the surgeon. Abbreviated and necessary procedures such as packing procedure only done to keep the patients alive are called "damage control surgery" (DCS). In contrast, prolonged and extensive surgical procedures performed on critically injured patients often results in poor outcome with high mortality rates of 46% and 80% for grade IV and V injuries, respectively [1]. The majority of these deaths (54%) have been attributable to hemorrhage with resulting coagulopathy, acidosis, and hypothermia [2]. Peri-hepatic packing (PHP) procedure, which is the basic damage control technique to arrest hepatic hemorrhage, is one of the cornerstones of the trauma surgery and currently, this is the most commonly accepted and performed method for major liver trauma. The main goal after packing is to correct acidosis, hypothermia, and coagulopathy, the lethal triad causing death [3]. The literature review has allowed for emphasis on the most common problems of PHP, adequacy of particular indications, their evolution, timing, the results in general and critical situations in particular [4].

This procedure requires caution during application, close observation after operation and experience to repair the injury in the re-look operation. Particularly, in some primary or secondary health care centers where a well-established intensive care unit or hepatobiliary or trauma surgeon is not available, the management of the trauma patients with severe hepatic injury is very difficult and mostly impossible. Thus, the damage control surgery, PHP for liver injury, has become the most common choice for temporary surgical treatment. Hence, we aimed to evaluate the outcome of our patients with PHP due to trauma and the points of caution that may affect the results and thus should be taken into consideration during PHP.

### Patients and methods

The trauma patients with liver injury admitted or referred to Ege University School of Medicine, Department of General Surgery between 2001 and 2006 were retrospectively evaluated. The patients who had undergone PHP to control hemorrhage due to major liver injury were included in the study. The demographic variables, injury mechanisms, associated injuries, blood transfusion requirement, the timing of packing removal, presence of additive interventions, and morbidity and mortality rates were reviewed.

An emergency laparotomy was performed on the patients with persistent hemodynamic instability despite both crystalloid and colloid replacement, an acute abdomen with the symptoms of peritonitis or prominent distension, ongoing blood transfusion requirements, penetrating abdominal injuries with confirmed peritoneal injury, and the finding of extensive free fluid on focused abdominal sonography for trauma (FAST). The criteria for inadequate packing included hemodynamic instability, low hematocrite values, even after the transfusion of many units of blood, insertion of an intraabdominal drainage catheter and signs of inappropriate packing procedure (the number of packs fewer than required or packs placed in wrong locations) in the re-look operation. The patients who had active hemorrhage from the drain placed in the first operation and persistent hemodynamic instability were submitted to an early operation. If there was enough time considering the Patient's hemodynamic criteria, routine computed tomography was also done to determine the extent of the injury.

All the patients were resuscitated according to Advanced Trauma Life Support (ATLS) recommendations [5]. During laparotomy, the liver injury was graded according to the liver injury scale of American Association of Surgery in Trauma [6]. The damage control surgery consisted of a midline laparotomy with minimal liver mobilization and an initial four-quadrant packing to investigate any additional intra-abdominal injury. On-going bleeding from the liver led to liver packing in conjunction with the Pringle maneuver and selective ligation of any visible bleeding vascular structure or injured bile duct. In such circumstances, the Pringle maneuver was used for a period of 20 min, after which the clamp was released and the liver was re-examined for any further bleeding. Failure to control bleeding led to packing the liver systematically with 5–8 abdominal swabs, considering the capacity of the intraabdominal cavity, in order to restore liver continuity and to provide compression. Temporary closure techniques were applied if there were any concerns about the intra-abdominal pressure.

All the patients were divided into two groups as Group A and Group B depending on whether the PHP procedure was adequate/appropriate or not, respectively. Inadequate or inappropriate packing procedure was based on the following criteria: i- placing the packs on only one side of the liver (anteriorly or posteriorly), ii- use of inadequate (underpacking) or excessive number of packs (overpacking) to maintain required pressure on the liver, iii- inserting a drainage catheter intraabdominally. Because the aim of packing is to maintain enough pressure as a mechanical compression to the liver and hematoma formation, the concept of the use of drains with packing is not correlated with the concept of ideal packing procedure and therefore the third criterion was enrolled to the study protocol. The patients in both groups were ultimately transferred to the intensive care unit postoperatively. A re-look laparotomy was performed when the patient's hemodynamic stability was provided.

The data were analyzed using SPSS 13.0 for Windows. Sample *t*-test was used for comparison of the two groups. A value of P < 0.05 was considered statistically significant.

## Results

Over a five-year period, 80 patients (M/F: 69/11) with a mean age of 34.3 years (range: 17–75 years were diagnosed as having hepatic trauma. Nineteen patients (23.7%) who had undergone PHP were detected. Thirteen of them (68%) were referred by other hospitals. Of these, 9 patients with inappropriate packing procedure were evaluated in Group B and the others (10 cases with adequate packing) were classified in Group A. Four patients subjected to intraabdominal drainage catheter and 5 patients because of improper location or inadequate number of the packs were evaluated in Group B. None of the patients with overpacking was observed. The demographic characteristics and associated injuries of the patients are shown in Table 1. The two groups did not significantly differ for associated injuries. Mean three units of blood were transfused in Group A, and unpacking procedure was performed in the 24^th ^hr, except in two patients, who were not proper for re-look laparotomy and had their first re-look in the 48^th ^hr. Only three (30%) patients required segment resection with homeostasis, and one patient underwent traumatic Whipple procedure and right hemicolectomy. This patient died in the postoperative day 10 due to septic complications and multiorgan failure. Another death was due to intracranial hemorrhage on the postoperative day 2. Radiological intervention was performed in a patient in Group A due to failure of packing to stop bleeding totally and it was successful. Transcatheter selective arterial embolization of the segment 7 was performed. In Group B, five patients (55%) required repacking in the first six hours due to remarkable deterioration of their general condition. Four patients had been placed an intraabdominal drainage catheter in the previous hospital before they were transferred to our institution. Mean 8 units of blood were transfused until unpacking procedure. Four patients (44%) required hepatic resection with homeostasis and two of them were observed with vascular injury to hepatic veins; right hepatic vein and retrohepatic accessory hepatic vein. One patient died from hemorrhagic shock intraoperatively and two patients succumbed to death postoperatively. Thus, a second operation could not be performed on these patients in our clinic (third operation overall). In the late period, one patient died due to sudden deterioration in his general condition and respiratory functions, which was diagnosed as pulmonary embolism. The information on the operations and postoperative period are provided in Table 2.

**Table 1:** The patient's demographic characteristics, intraoperative and postoperative observations.

	**Group A**	**Group B**	**Total**
**Patient number**	10 (%52)	9 (%47)	19/80 (%23.7)
**Age (mean, range)**	53.5 (18–75)	44 (23–60)	56 (18–75)
**Gender (M/F)**	8/2	7/2	15/4
**Internal/External Hospital**	6/4	0/9	4/15
**Associated organ injury**			
**No**	5	3	8(%42)
**Visceral organ**	3*	1(spleen)	4(%21)
**Others**	2**	5**	7(%36)

*kidney, pancreas, small intestine, colon

**intracranial or intrathoracic trauma, rib or extremity fracture,

**Table 2:** Intraoperative observations and outcome

	**Group A(n = 10)**	**Group B(n = 9)**	**Total**
Grade of injury (IV, V, respectively)	6, 4	5, 4	11, 8
Mean blood transfusion (unit)*	3 (2–6)	8 (7–12)	6,2 (2–12)
The timing of packing removal (days, 0/1^th^*/2^nd^) (patient number, respectively)	1/8/1	4,2,3	5(26%), 10(52%), 4(21%)
Surgical procedure			
Simple saturation & homeostasis	7	6^α^	13(68.4%)
Segmental resection	3	4^γ^	7(36.8%)
Additive procedures	2	3	5(26.3%)
ICU stay, days (mean, range)*	10,7 ± 2 (8–16)	15,6 ± 4 (11–22)	
Mean length of hospital stay, days	30,7 ± 8 (18–49)	36,1 ± 9(23–51)	
Postoperative mortality	2(20%)	4(44%)	6(31.5%)

ICU: intensive care unite, ^α ^: one of them died intraoperatively, ^γ^: two of them underwent vascular repair,

• P value < 0.05

Considering the time for pack removal in the first 24 hrs, the number of patients who were proper for second intervention was statistically higher in Group B than in Group A (55% versus 10%, respectively). Majority of the patients (90%) in Group A were performed pack removal and surgical repair after the first 24 hrs (p < 0.01). The length of intensive care unit stay was significantly lower in Group A (p = 0.021). The length of hospital stay, however, was not significantly different but relatively lower in Group A than in Group B. Overall morbidity rate was 47.3% (9/19 patients). Postoperative complications included pneumonia (4 patients; 21%), pleural effusion (4 patients; 21%), intraabdominal fluid collection (4 patients; 15.7%); one in Group A and three in Group B, wound site infection (3 patients; 15.7%), and sepsis (2 patients; 10%). Intraabdominal fluid collection was treated by percutaneous drainage in three patients and spontaneous resolution was observed in the last one. Two of four cases had concomitant hollow organ injury. Overall mortality rate was 31.5 % (6/19 patients). The causes in Group A included head trauma (n = 1) and septic complication (n = 1); in Group B, hemorrhagic shock (n = 2) (death on the operating table), multiorgan failure (n = 1), likely due to hypovolemic shock and hypoperfusion of the organs, and postoperative septic complication (n = 1).

## Discussion

Many major liver injuries and progressive coagulopathy are the most frequent indications for DCS, and surgical experience is very important during this process [7]. The "damage control" concept has been shown to increase overall survival and is likely to modify the management of the critically injured patient. Pringle maneuver, which is one of the damage control surgeries in normothermia, is also safe for at least 60 minutes; this was maintained up to 85 minutes [8]. However, because the tolerance of the liver to hypoxia decreases in such trauma patients who are susceptible to hemorrhagic shock, the time of occlusion is kept as short as possible. In our series, the Pringle maneuver was used for a period of 20 min, after which the clamp was released and the liver was re-examined for any further bleeding. Selective ligation of the hepatic artery in the patients with any arterial bleeding was then undertaken, or other definitive surgical interventions were performed. It is important to recognize that liver packing will not control arterial bleeding and that any bleeding artery should be suture-ligated before packing procedure. However, a significant number of the patients with high-grade liver trauma tend to develop consumption coagulopathy, in which case none of the surgical procedures can be successfully performed. Additionally, when the bleeding is mainly from retrohepatic veins or the portal vein, hepatic artery ligation alone will fail to control the bleeding. In such circumstances, perihepatic packing, a definitive hemostatic procedure, is currently a well-accepted technique for severe liver trauma when routine procedures cannot control the bleeding. The main aim of PHP, one of the lifesaving part of the DCS, is to overcome the lethal triad correcting the acidosis (pH < 7.2), hypothermia (<35°C) and coagulopathy (prothrombin time >16s). For many years, therefore, PHP has been one of the most popular methods for the management of major liver injuries. The description of this method comprises several parts [9]. The initial operation, which should be performed minimally, essentially includes the control of exsanguinations, ligation of active bleeders or more complex procedures including hepatectomy, and prevention of contamination of the peritoneal cavity. After a further resuscitation progress in the intensive care unit characterized by improved hemodynamics, correction of coagulopathy, rewarming, and complete ventilatory support the patient will be ready for re-look operation [10]. It enables the recognition of the main injury after the removal of the packs and a definitive surgical repair of the injury. In the present study, three patients did not have a chance to undergo a second operation in our institution; one of them died from severe exsanguination during the first operation, and the other two patients succumbed to death in the intensive care unit in spite of administration of triple inotropic agents accompanied by supplementary drugs after the first operation in our institution. The last two cases had lost excessive amount of blood due to underpacking and other injuries. In Group B, the mean unit of blood transfusion (3 units vs. 8 units, p < 0.05) and mortality rate were higher, but only the difference between the mean blood units transfused was statistically significant. Transfusion of many units of blood due to continuing high blood loss might slow down the coagulation cascade resulting in disseminate intravascular coagulopathy and can also lead unpreventable severe acidosis [11]. In such conditions, PHP is a lifesaving technique for temporary control of severe liver injury providing enough time to correct either physiological or metabolic derangements.

The most common problem with this technique is the determination of timing for the placement and removal of the packs and the method which should be used [4]. The decision to pack should be made early in the exploration in order to provide better chance of survival for liver trauma patients. MacKenzie et al reported a trend toward avoidance of complex surgical maneuvers in the initial operation but an early re-look surgery [12]. The principal indications are as follows: the patients with complex anatomic lesions accompanied by uncontrollable hemorrhages, especially consumption coagulopathy, and unstable patients who develop hemorrhagic shock are, therefore, applied PHP to minimize the impact of prolonged shock. To manipulate the liver easily, the ligamentous attachments (falciform, right triangular, and coronary) should be divided to allow any intervention to the right lobe, which is the mostly affected part of the liver. On the other hand, it is better not to divide the hepatic suspensor ligaments, only if packing procedure is decided to perform. The critical point is that turning the liver over for examination of the retrohepatic vana cava should be avoided; otherwise, life-threatening exsanguinations will not be a surprise. Additionally, the liver should never be sutured over a bleeding vessel, especially when a deeper injury is suspected. It may be unavoidable to experience a posttraumatic hemobilia, which may develop due to intrahepatic hematoma with biliovascular fistulae. The second important thing is that an intraabdominal drainage catheter is incompatible with the concept of the PHP. This process does not allow for the development of hematoma which is necessary to stop bleeding. In addition to these, the number of packs should be well-matched to the estimated volume of the patient's intraabdominal cavity considering the body weight and height. The evaluation of the patient's ribs with palpation to determine appropriate pressure can also be efficient. On the other hand, the organs, particularly the kidneys (pressure on the inferior vena cava) and lungs (pressure to the diaphragm), could be exposed to over pressure, and this may result in organ failure due to abdominal compartment syndrome. Gao et al reported a rate of 10% for abdominal compartment syndrome in the packed patients [13]. According to our criteria, sufficient number of packs (6–9 numbers) should be placed on both the anterior and posterior surface of the liver, never into the laceration. This can cause to the enlargement of the injury site. In our series, the number of the packs varied between 5 and 8. However, the surgeon's intraoperative observation is the key-point for this detail. Compressing maneuver can only maintain the appropriate pressure of the liver. It can also be temporarily performed at the time of operation and involve pushing back and up (spine and diaphragm) the liver to stop the hemorrhage. The liver packs should be removed as soon as the patient is stable considering the components of lethal triad [14-16].

Early recognition of the failure to control the hemorrhage and good timing of the pack removal are the cornerstones of success [17]. However, the time of removal of the pack is still a controversial problem related to PHP. Although, there is still no consensus on this challenging issue, in general, this time should be determined considering the required duration to achieve the main goal of the PHP: correcting lethal triad [3]. Because, if lethal derangements, which may occur very fast in the trauma patients, become established, a vicious cascade, almost impossible to overcome, is formed. The optimal time to overcome the lethal triad and re-exploration is generally reported as 24–48 hrs. Various authors have reported different time intervals for re-look operation and removal of packs. This usually takes 12–36 hrs to achieve, yet liver packs have been removed as long as 7 days after the initial packing [18]. Caruso et al. demonstrated that re-bleeding from the liver was greater when liver packs were removed within 36 hrs than after 36 hrs [19]. Likewise, Nicol et al reported a significantly higher rate of bleeding when the packs were removed at 24 hrs than at 48 hrs [20]. On the other hand, Krige et al stated that after 3 days, the risk for intra-abdominal sepsis became greater (83%) [21]. On the contrary, Nicol et al pointed out that there was no association between the total duration of packing and the development of liver-related complications or intra-abdominal collections, but the presence of concomitant small bowel or colon injury to liver and open abdomen after PHP were found as important factors with regard to the development of an intra-abdominal collection [20]. The routine time for the removal of the packs, after the first 24 hours in our institution as accepted by most centers, could not be practiced in Group B. The time for the removal of the packs in two groups was statistically significantly different. Nevertheless, in the present study, the timing of pack removal for the majority of the patients in Group B was <24 hrs due to inappropriate/inadequate packing. In Group A, despite appropriate PHP procedure, early removal of the packs was required in only one patient. Likewise, in Group B, one of the patients who had been performed PHP with placement of a drainage catheter containing approximately 250 cc blood required another laparotomy within the first 6 hours. Selective hepatic artery ligation with re-PHP procedure was applied in the re-look laparotomy. Intraabdominal packing could not stop all the hemorrhages, and thus, 10.5% of the patients exsanguinated. Sharp et al noted a rate of 23% for failure in packing to achieve hemostasis [18]. Combinations of surgical and radiological interventions should be performed to reach best outcome. Angiography and hepatic embolization are used to be performed in those patients in whom the second attempt to remove the liver packs was unsuccessful because of liver bleeding [20] or in whom complex liver injuries were observed in unstable patients [13]. However, recently, combinations of surgical and radiological interventions have become popular because of its superiority over definitive surgery that is used in the first operation. Hepatic angioembolization has been recommended immediately post-packing, and certainly this may prove to be a useful adjunct in controlling hepatic hemorrhage [13,22]. In one of the patients in Group A, radiological intervention was performed due to failure of packing alone to stop bleeding totally and it was successful.

The prognosis of the patients with severe liver injury depends on both the efficacy of the PHP and the presence and severity of associated injuries. Survival rate in Group A (80%) was well-matched to the literature (56–82%) [10,18,23], whereas it was lower but not statistically significant in Group B (56%). It should be emphasized that if PHP is inadequately performed as "underpacking", it causes immediate failure due to ongoing hemorrhage. Likewise, if it is excessively performed as "overpacking", it may lead to abdominal compartment syndrome or multiorgan failure. Thus, if it is indicated and a decision is made to perform PHP, it should be done in an order by placing the proper number of packs into the right locations, and no intraabdominal drainage catheter should be used. Determining the right time to remove the packs and appropriate surgical procedure also has an important effect on patients' outcome. In the light of our results, it can be concluded that inefficient and inappropriate PHP procedure results in poor outcome requiring more units of blood transfusion, earlier re-look laparotomy, and longer intensive care unit stay.
